# Is there any gender-specific impact in the treatment of patients with basal cell carcinoma in the head and neck region?^✰^

**DOI:** 10.1016/j.jpra.2023.09.012

**Published:** 2023-10-05

**Authors:** L. Hartlieb, P. Funovits, W. Pipam, M. Rab

**Affiliations:** a Department of Plastic, Aesthetic and Reconstructive Surgery, Klinikum Klagenfurt am Wörthersee, Klagenfurt am Wörthersee, Austria; b Center for Interdisciplinary Pain Therapy, Oncology and Palliative Care, Klinikum Klagenfurt am Wörthersee, Klagenfurt am Wörthersee, Austria

**Keywords:** Basal cell carcinoma, Head and neck, Surgical excision, Gender, Outcome, Defect coverage

## Abstract

**Background:**

There are no current studies concerning gender-specific impact on the treatment of BCCs. We performed a retrospective analysis with the aim of showing that selection of treatment by physician and patients’ evaluation concerning quality of life and aesthetic outcome has a gender-specific impact.

**Methods:**

Overall, 47 patients treated by excision of BCC from the head and neck region at our department from 2015 to 2020 were included. Defects were closed via flap, split-thickness skin graft. or primary closure. Pain, scar quality, patient satisfaction and quality of life were ascertained by the Skin Cancer Index (SCI), the Basal and Squamous Cell Carcinoma Quality of Life (BaSQoL) questionnaire, Patient and Observer Scar Assessment Scale (POSASv2.0EN) and Vancouver Scar Scale (VSS).

**Results:**

Women received significantly more flaps than split-thickness skin grafts (*p* = 0.025). The coverage method was independent of surgeons’ gender.

Patient's POSAS were higher in women (*p* = 0.087), and observer's POSAS (*p* = 0.229) and VSS (*p* = 0.7) showed no significant difference between genders.

SCI and BaSQoL scores showed that women are significantly more critical than men after BCC treatment (SCI *p* = 0; BaSQoL *p* = 0.022). Furthermore, dermatological follow-up frequency was significantly higher in women (*p* = 0.035).

**Conclusion:**

We determined the gender-specific impacts on the treatment of patients with BCCs regarding methods of closure, post-interventional dermatological follow-ups, quality of life, scar quality, and overall patient satisfaction. No difference in scar quality was found when assessed by physicians.

## Introduction

Basal cell carcinoma (BCC) is the most common type of skin cancer in Caucasians, and predominantly arises in the head and neck region. Although BCC metastasis is rare, it can induce extensive local tissue damage and is associated with significant morbidity and mortality in elderly adults. BCC mainly occurs in patients after their 5th decade of life, with a female: male ratio of 2:1. Recurrences are common (2–8% in BCCs treated with surgical excision),^1^ especially in predisposed patients.

The most significant environmental risk factor for the development of BCC is sunlight exposure. Ultraviolet B (UVB) radiation is the main carcinogenic factor.^2^^,^^3^

Other risk factors include Fitzpatrick skin types I and II, immunosuppression, and genetic predispositions, such as Xeroderma pigmentosum, Gorlin–Goltz syndrome, Rombo syndrome, and Bazex–Dupré–Christol syndrome.^4^^,^^5^

According to histopathological characteristics, there are several BCC subgroups with increasing invasiveness from superficial, nodular, morphoeic, and basosquamous tumors. Approximately 60% of all BCC cases present with the nodular type of lesion.^3^

Due to the fact that this tumour predominantly arises on facial skin, its removal, prevention of recurrent tumors, preservation of healthy skin, aesthetic outcome, and financial costs are important factors to consider in the treatment process.

Worldwide, the treatment of choice for BCC is surgical excision.

However, reviewing the current literature, there is a lack of studies reporting a comparison of the gender-specific impact in the treatment of patients with BCC in the head and neck region.

The aim of this study was to evaluate gender-specific impact on the surgeon's selection of treatment and subsequent gender-specific assessment of perceived outcome by the patient.

## Materials and methods

### Patient recruitment

All patients treated by R0 surgical excision of diagnosed and histologically verified BCC at our department from 2015 to 2020 were included in the study. Therefore, limitations were not included in terms of the sex and age of patients. Patients who were histologically diagnosed via frozen section were excluded from the study. Patient examination was performed by a male and a female examiner. Twenty-six men and 21 women with an average age of 72.72 years (range, 50–89 years) were included in the study.

### Surgical technique

Surgeries were performed by 10 experienced senior plastic surgeons, 2 female and 8 male. Overall, 9 patients were operated on by women and 38 by men. After sterile washing, covering, and local anaesthesia application, the lesion was surgically excised. The defect was temporarily covered using EpiGARD^Ⓡ^ (BIOVISION GmbH) skin replacement, which can be left on the defect for up to 7 days. Further resection was performed if a narrow-wound edge was found in healthy tissue. After definitive R0 histological finding, the skin replacement was removed and the defect was closed either with a flap, a split-thickness skin graft, or by primary closure, depending on the size and region of the defect. Split-thickness skin grafts were used in large defect-sizes and primary closure in small defect-sizes. Flaps were mostly used for defects in the nasal unit for better aesthetic outcomes and to prevent deformation of tissue caused by primary closure in this region.

### Follow-up

Follow-up examination was performed on an average of 3.7 years (range, 1–7 years) after intervention by the same examiners, which included one male and one female examiner. The gender-specific impact on both the patients and evaluating physicians was examined. Baseline data, pain, scar quality, patient satisfaction, and quality of life were ascertained using 4 questionnaires: The Skin Cancer Index (SCI),^6^ basal and squamous cell carcinoma quality of life (BaSQoL)^7^ questionnaire, patient and observer scar assessment scale (POSAS v2.0/EN),^8^, ^9^ and Vancouver Scar Scale (VSS).^10^ The validated SCI, a self-assessed questionnaire measuring health-related quality of life (HRQL) in patients with cervicofacial non-melanoma skin cancer (CFNMSC) consists of 15 questions, which query about personal view of skin cancer, its treatment, and impact on social life, work life, home life, and other areas of concern. The valuation is based on a scale from 0 (not at all) to 5 (very much).

The BaSQoL questionnaire, also reflecting quality of life after treatment from the point of view of the patients, consists of 16 items. Each item can be answered within a scale from 0 (not at all influencing daily life) to 3 (very much influencing daily life). The patient and observer scar assessment scale (POSAS) is a valid scar and pain assessment scale that measures scar quality from 2 perspectives of the patient and clinician. Both, the patients and the clinicians’ questionnaire form comprise 6 items (vascularity, pigmentation, thickness, relief, pliability, and surface area).

All items are scored on a scale ranging from 1 (such as normal skin) to 10 (worst scar imaginable).

The Vancouver Scar Scale is evaluated and filled in by the physician to assess and quantify the severity of an abnormal scar. Parameters such as vascularity, pigmentation, pliability, height, pain, and itchiness are evaluated. Each parameter contains ranked subscales that may be summed up to obtain a total score ranging from 0 (normal skin) to 18 (worst scar imaginable).

The dermatological follow-up examinations after BCC diagnosis were also itemized by gender and number of checkups per year.

### Statistical analysis

The data were evaluated using SPSS Version 18.0 for Windows. Descriptive statistics were used to describe the samples. Simple frequency comparisons were calculated using chi-square, comparisons of means between study groups were performed using the *t*-test for independent samples. The significance level was set at *p* = 0.05 (5% level).^11^

## Results

### Patient data

A total of 47 patients between the ages 50 to 89 years, with a mean age of 72.72 years were enroled in this study. Analysing the age of these patients in percentiles revealed that our study population was comprised of older adults (percentile 25: 68.00; percentile 50: 75.00; and percentile 75: 80.00). The mean follow-up time was 3.7 years (range, 1–7 years).

For defect coverage, 29 (61.7%) patients received different types of flaps, in 12 (25.5%) patients split-thickness skin graft was used for defect coverage, and 6 (12.8%) excision sites were closed primarily.

Out of the 29 patients who received flaps, 16 (55.2%) were women and 13 (44.8%) were men. In the group that received split-thickness skin grafts for wound closure, 2 patients (16.7%) were women and 10 (83.3%) were men.

There was no statistically significant difference in the gender of the physician who advised further ongoing treatment for the patient.

### Recurrence and number of surgeries

None of the patients showed BCC recurrence. During the follow-up period, only one patient showed a new BCC occurrence in a different location (in the face).

The mean number of surgeries was 2.7 times, 22 (46.8%) patients were operated on twice (Table 1). The higher number of surgeries were from further resections due to narrow-wound edge in healthy tissue.

**Table 1:** Number of surgeries.

	Total	Women	Men	Flaps	Skin grafts	Primary closure
1x	3	1	2	0	1	2
2x	22	11	11	13	7	2
3x	10	6	4	9	1	0
4x	8	3	5	5	2	1
5x	3	0	3	3	0	0
>5x	1	0	1	0	1	0
Mean:	2.79	2.52	3	2.97	2.75	2

### Questionnaires for scar quality, aesthetics, and quality of life

Regarding scar quality and aesthetics, the VSS showed a mean score of 3.6 in women and 3.7 in men, with a maximum score of 18. There was no significant difference between men and women (*p* = 0.7; Table 2).

**Table 2:** Questionnaires for scar quality and aesthetics.

	POSAS patient	POSAS observer	VSS
Mean score women (flap + skin graft)	12.06	13.41	3.59
Mean score men (flap + skin graft)	8.65	10.96	3.70
Mean score women (flap)	11.2	11.87	3.8
Mean score men (flap)	8.38	9.92	5.92
Mean score women (skin graft)	18.5	25	2
Mean score men (skin graft)	9	12.3	3
Mean score women (primary closure)	7.67	9	2
Mean score men (primary closure)	11	9.67	3

The other questionnaire used for scar quality and aesthetics was the patient and observer scar assessment scale (POSAS). The patient scores for women were higher than those for men (women: 12.06; men: 8.65), with a trend toward statistical significance (*p* = 0.089), whereas the observer scores were slightly higher for men (women: 13.41; men: 10.95). Additionally, the observer scores were higher than the patient scores. Scores for defects covered via primary closure were excluded. For the POSAS observer score, there was no significant difference (*p* = 0.229; Table 2).

BaSQoL questionnaire and SCI were used to assess the quality of life. The mean score for women (BaSQoL: 11.2; SCI: 19.74) was significantly higher than that for men (BaSQoL: 4.70; SCI: 5.31) in both questionnaires with a high significance in the SCI (*p* = 0) and BaSQoL (*p* = 0.022; Table 3).

**Table 3:** Questionnaires for the quality of life.

	Mean score women	Mean score men
SCI	19.74	5.31
BaSQoL	11.2	4.70

### Defect-size and BCC regions

With reference to the defect size (Table 5), flaps were always performed on a defect larger than 0.32 cm^2^ and split-thickness skin grafts on a defect size larger than 2.60 cm^2^. The mean defect sizes in patients receiving flaps and split-thickness skin grafts were 3.54 and 12.93 cm^2^, respectively.

The BCCs occurred mostly on the nasal unit (21 cases; 44.7%), followed by the forehead unit (15 cases; 31.9%), around the ocular region (3 cases; 6.4%), and on the cheeks, lip region, ear region, or on multiple sites (2 cases each [4.25%]; total 17%; Table 4).

**Table 4:** Number of BCCs in different regions.

Head and neck regions with BCC occurrence			
	Total	Women	Men
Nasal unit	21	12	9
Cheek unit	2	1	1
Forehead unit	15	6	9
Lip region	2	1	1
Ocular region	3	1	2
Ear region	2	0	2
Multiple regions	2	0	2

**Table 5:** Surface of the sample / defect size.

Surface of the sample (cm^2^)						
Defect-size skin grafts (cm^2^)				Defect-size flaps (cm^2^)		
	Total	Women	Men	Total	Women	Men
Mean	12.94	18.54	11.81	3.54	4.39	2.40
Min.	2.6	12.58	2.6	0.32	0.80	0.32
Max.	32.50	24.50	32.50	27	27	6.60

We also examined how often patients with BCC diagnosis and treatment visit the dermatologist for follow-up appointments. Frequency of dermatologic consultation for follow-up check-ups was more than 3 times higher in women than in male patients with a statistically significant difference (*p* = 0.035), (Table 6).

**Table 6:** Dermatologist appointments per year.

Dermatologist appointments per year		
Mean	Women	1.67
	Men	0.52
	Total	1.03

### BCC histology

Histological studies indicated that the solid trabecular type (*n* = 17) variant of BCC occurred most often, followed by mixed BCC-subtypes (*n* = 14). Other types of BCC are listed in Table 7.

**Table 7:** BCC-subtypes.

BCC-subtypes	
Solid trabecular BCC	17
Mixed subtypes	14
Ulcerated, solid trabecular BCC	6
Sclerosing BCC	4
Nodular BCC	2
Multicentric BCC	2
No histological subtype cited	2

## Discussion

BCC most often occurs in the head and neck region, leading to consecutive tissue destruction. Due to the fact, that surgical excision is the gold standard for BCC therapy worldwide, a residual visible scar, beside R0 excision is expected in all cases. Several publications deal with different types of defect coverage after tumour excision.^12^, ^13^, ^14^

However, none of these publications focus on effects of gender distribution in BCC treatment. The present study revealed that most patients (29; 62%) underwent a flap coverage of the defect after BCC excision. In this cohort, 16 patients were women (55%) and 13 were men (45%). In the group of patients who were treated with split-thickness skin graft (12; 25.5%), 2 were women (17%) and 10 were men (83%). Review of current literature showed that our results that demonstrate the distinct impact of gender distribution in BCC treatment are being reported for the first time to our knowledge.

The impact of the patient's gender on the examining physician was found to be statistically significant (*p* = 0.025), showing unequivocally that women were treated in almost all cases with a flap and only rarely with a split-thickness skin graft. However, the split-thickness skin grafts cohort was small in this study. After reviewing the literature, the impact of gender distribution on the type of BCC treatment in the head and neck region has been reported for the first time. Furthermore, this study reveals that surgeons tend to treat women more with flaps, hoping to attain the best aesthetic outcome, than with split-thickness skin grafts. Surgeons in our department tended to apply more split-thickness skin grafts than flaps in men. Considering these findings, the impact of the patients’ gender showed a statistical significance on treatment selection by the physician, whereas the choice of treatment was independent of the gender of the examiner (Table 8).

**Table 8:** Pairing gender of surgeons to gender of patients and kind of surgery.

	Flaps		Split-thickness skin grafts		Primary closure	
	Male patient	Female patient	Male patient	Female patient	Male patient	Female patient
Male surgeon	10	12	8	2	3	3
Female surgeon	3	4	2	0	0	0
Total	13	16	10	2	3	3

POSAS score, which divides a patient from an observer, revealed that women have more impact than men on the aesthetic outcome. Regarding scar formation, women score more critically than men, with a trend toward statistical significance (*p* = 0.089). Such gender-specific and increased critical self-evaluation of the postoperative aesthetic outcome has already been recorded in literature.^15^^,^^16^

However, no statistically significant difference (*p* = 0.229) was found when comparing the gender impact in scar quality scoring as judged by the physician.

These findings could be confirmed by the results from the VSS questionnaire, which was evaluated by clinicians as well. This score showed no significant difference in the scar quality between genders (*p* = 0.7). This might be explained by the fact that surgeons evaluate scar quality and aesthetic outcome, owing to their medical approach and knowledge, in a very precise, different, and more objective way compared to that by the patients.

Regarding POSAS score from the physician's point of view, a statistically significant difference in aesthetic outcome could be detected when comparing gender specific results of flaps and split-thickness skin grafts in all patients (results of men and women combined). From the patients’ point of view (refer to POSAS score patient view), no statistically significant difference could be detected. Furthermore, the results show that there is a tendency toward statistical significance (*p* = 0.071) between the group of patients treated with a flap and those with split-thickness skin graft, in favour of the patients who received a flap.

The results further show that women with a BCC diagnosis are more likely to evaluate quality of life more critically than men. A statistically significant difference could be shown in the two applied questionnaires (SCI: *p* = 0; BaSQoL: *p* = 0.022). This finding is in accordance with current literature.^16^

After diagnosis and planning of therapy, men seem to be less concerned about follow-up of therapy than women. This could also be confirmed by the frequency of check-ups at dermatologist. On an average, women tended to make three times more appointments at the dermatologist than did men after BCC diagnosis and subsequent surgery (*p* = 0.035).

In terms of patients’ demographics, BCCs occurrence might correlate to the older age of the patients in this study. Asgari MM et al. stated that BCCs are most frequently seen after the age of 50 years.^17^ This is in accordance with our findings, where the mean age of patients was 72.72 years.

In conclusion, women are more critical than men, when assessing quality of life and aesthetic outcome after BCC excision and defect coverage. These findings might influence us to use flaps more frequently in women than in men. The question remains open whether the less critical male gender is prone to receiving more split-thickness skin grafts with significantly worse aesthetic outcomes, whereas the more critical female gender tends to receive more time-consuming flap procedures with better aesthetic outcomes. In terms of follow-up, women have a statistically significant higher awareness for screenings by the dermatologist than men.

## Conclusion

There is a gender-specific impact on the selection of treatment by physicians: Women tend to receive significantly more flaps for head and neck BCC than men. On the other hand, there were no statistically significant differences found in the evaluation by the physician, irrespective of whether the physician was female or male.

Choice of skin graft and flap was dependent on defect size and gender.

Another gender-specific impact is observed on postoperative follow-up at the dermatologist after BCC diagnosis and treatment. Women consulted the dermatologist 3 times more often than men with a statistically significant difference (*p* = 0.035).

Furthermore, a statistically significant difference was found in the results of all self-administered questionnaires that showed that women are more critical than men about the quality of life and aesthetic outcome after BCC diagnosis and treatment.

Regarding patient satisfaction, quality of life, and aesthetic outcome, women were significantly more critical than men.

### Limitations

The study is limited by its retrospective nature. Patients were examined in a mean follow-up period of 4 years, which can influence the assessment of the questionnaires used. Furthermore, only a limited number of patients received the split-thickness skin grafts. We would recommend further investigations in controlled multicenter settings with a higher population. Therefore, we have started a prospective data collection for future research.

Ethics: The research protocol was approved by the local Ethical Committee “Ethikkommission des Landes Kärnten”. S2023-03

## Conflict of Interest

None.
